# Gentamicin-permeated cement to sustain mechanical support for the treatment of a chronic calcaneal abscess. A case report

**DOI:** 10.1016/j.ijscr.2023.108846

**Published:** 2023-09-16

**Authors:** Kaissar Yammine, Bilal Alqaysi, Jad Mansour, Joeffroy Otayek, Jimmy Daher, Chahine Assi

**Affiliations:** a Department of Orthopedic Surgery, Lebanese American University Medical Center-Rizk Hospital, Lebanese American University, School of Medicine, Lebanon; b Diabetic Foot Clinic, Lebanese American University Medical Center-Rizk Hospital, Beirut, Lebanon; c Center for Evidence-Based Anatomy, Sport & Orthopedics Research, Lebanon

**Keywords:** Osteomyelitis, Diabetic foot infection, Cement, conservative surgery

## Abstract

**Introduction and importance:**

Chronic calcaneal osteomyelitis is a challenging condition associated with high relapse rates, requiring a multidisciplinary approach and various therapeutic options for effective management. We report a very rare case of a pyogenic osteomyelitis of the os calcis presented as a bone abscess.

**Case presentation:**

A diabetic male patient presented with chronic osteomyelitis of the calcaneus in the form of bone abscess with a cavity of 6*5 cm. After pus evacuation and debridement of the cavity, gentamycin-impregnated polymethylmethacrylate cement was used to locally assist in controlling the infection and to assure mechanical support. Antibiogram-based oral antibiotic was administrated for 6 weeks. At final follow-up, the patient could walk without any assistance and was able to raise his body on the operated heel, with no signs of infection.

**Clinical discussion:**

This case illustrates successful conservative surgical treatment of calcaneal abscess using antibiotic-impregnated cement for mechanical support and local infection control.

**Conclusion:**

Incorporating antibiotic-impregnated cement into conservative foot surgeries for deeply embedded calcaneal abscesses provides effective infection control, mechanical support, and functional preservation, leading to successful treatment outcomes.

## Highlights

• Chronic osteomyelitis of the calcaneus in the form of bone abscess is very rare.
• After pus evacuation and debridement, the cavity was filled with cement
• Besides local infection control, the cement was used to assure mechanical support.
• Antibiogram-based oral antibiotic was administrated for 6 weeks.
• At final follow-up, the patient was able to raise his body on the operated heel.

## 1 Introduction

Chronic calcaneal osteomyelitis is hard to manage and is associated with high relapse rates [1]. With an incidence ranging between 3 % and 10 % of all osteomyelitis, recurrence was reported as high as 40.5 % needing multiple and different treatments strategies [1]. Its treatment requires a multidisciplinary approach with various therapeutic options, such as culture-based antibiotics, surgical debridement, partial/total calcanectomy, flap coverage, or below-knee amputation [2]. Implementation of local antibiotic release systems by means of antibiotic-impregnated cement has been described as an effective treatment regimen [3]. Very few studies reported the use of cement as a spacer to treat a diabetic calcaneal osteomyelitis but none to our knowledge, described cement use in treating chronic calcaneal abscess. The current study presents a case of a diabetic patient with a chronic calcaneal abscess who was treated by evacuating the pus, debriding the bone, and filling the residual cavity with gentamicin-infused cement, providing mechanical support and local infection control.

This case report has been reported in line with the SCARE criteria [4].

## 2 Case presentation

A 65-year-old man with a 10-year history of type 2 diabetes mellitus presented to our hospital due to left heel swelling and persistent pain during rest and ambulation, which had been ongoing for 2 years. He had previously undergone primary Achilles tendon repair, which resulted in early rupture and subsequent revision surgery with tendon reinforcement using two anchors in the superior aspect of the calcaneal bone. Approximately ten months prior to presentation, a fistula developed at the wound site, accompanied by pus discharge. Despite receiving empiric oral antibiotics, the patient's symptoms and signs remained unchanged.

Physical examination revealed hindfoot swelling, a 5 mm opening along the distal Achilles scar, and normal ankle range of motion. The patient had a body temperature of 37.8 °C. Laboratory tests indicated a white blood cell count of 11,400/μl, fasting glucose level of 116 mg/dl, and HbA1c level of 11 %. It is of importance to note that the patient had a morbid obesity with a BMI of 42.8.

**Fig. 1:**
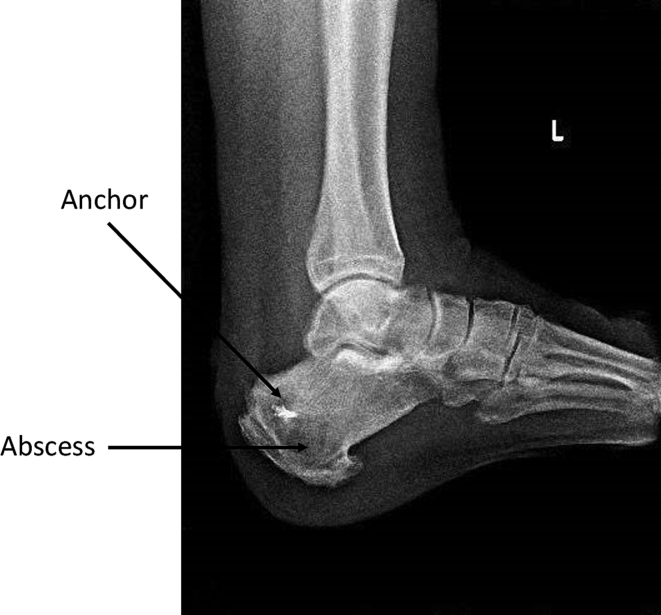
Pre-operative radiographs.

**Fig. 2:**
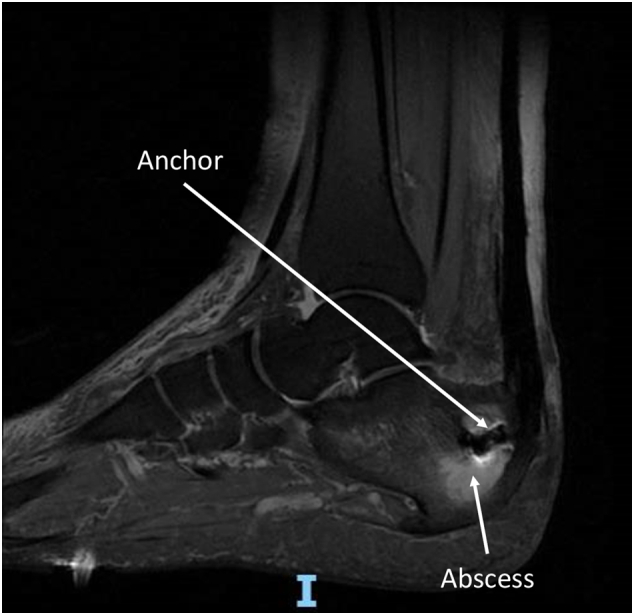
Pre-operative MRI.

Preoperative radiographs revealed a 6*5 cm cyst located distal and ventral to the anchors (Fig. 1). Magnetic resonance imaging confirmed the presence of a well-defined intraosseous cystic lesion at the calcaneal tuberosity, caudal to the surgical anchor, displaying peripheral rim enhancement and the characteristic “penumbra sign,” consistent with an intraosseous abscess (Fig. 2). The remaining os calcis exhibited diffuse bone marrow edema extending to the subcutaneous tissues. Based on these findings, the patient was diagnosed with chronic osteomyelitis in the form of a calcaneal abscess.

**Fig. 3:**
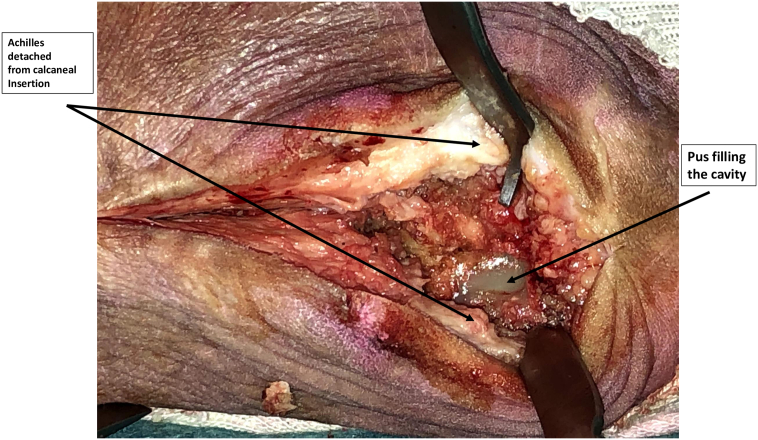
Intra-operative view.

**Fig. 4:**
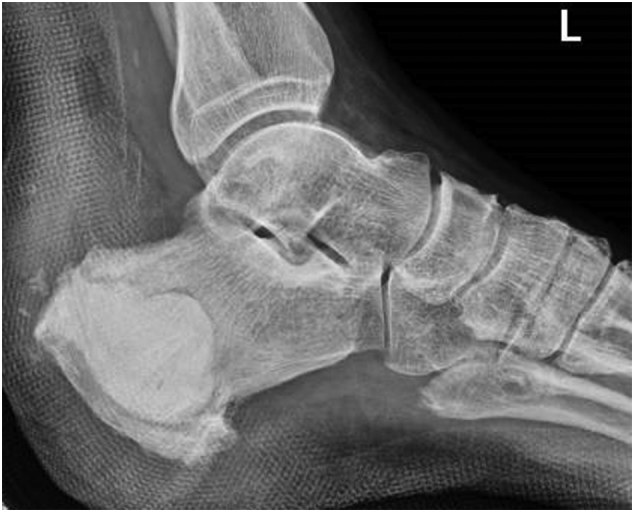
Post-operative radiographs with cement filling the cavity.

Under regional anesthesia, an incision was made at the distal insertion of the Achilles tendon, following a straight path deep to the midline axis. The Achilles tendon was then split down to the bone, and its attachment to the posterior aspect of the os calcis was detached both medially and laterally. On the medial aspect of the posterior os calcis, a 1 cm × 1 cm window was created, from which significant amounts of pus were drained under tension and subsequently sent for culture (Fig. 3). Utilizing the guidance of MRI images, the wall of the remaining cavity was completely excised using curettes and a small burr, with the assistance of fluoroscopy. Following a thorough irrigation, one ampoule (40 g) of a commercially prepared gentamicin polymethylmethacrylate (PMMA) cement (SmartSet GHV, Blackpool, England) was prepared and implanted within the cavity (Fig. 4). The concentration of gentamicin was 1 g. Assurance of complete filling of the cavity was confirmed prior to closure.

Proximally the Achilles split was closed by direct suture. The Achilles insertion was sutured to the surrounding soft tissues without any attempt to anchor its distal stump to the bone. Subsequently, a posterior splint was applied with the ankle positioned at 25° of plantar flexion.

**Fig. 5:**
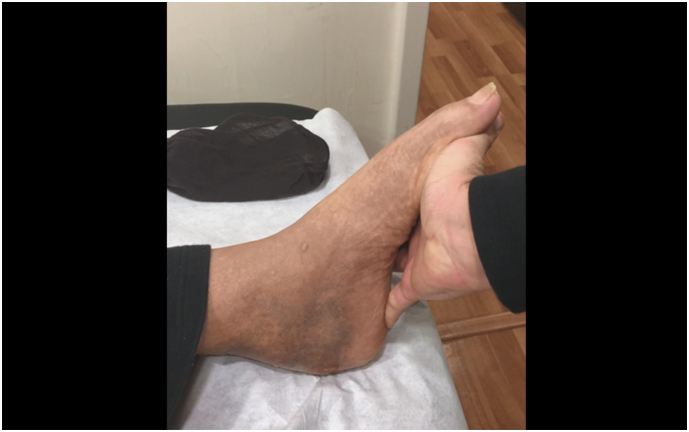
Excellent counter-resisted plantar flexion.

**Fig. 6:**
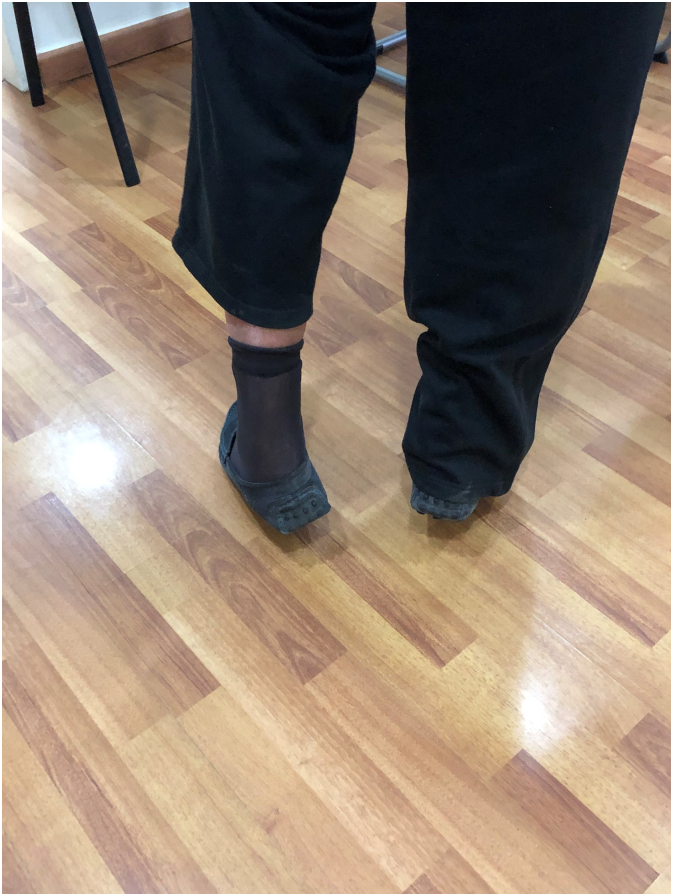
Standing heel raise test.

The patient was initiated on a systemic daily regimen of 2 g of Imipenem and 400 mg of Teicoplanin, which was continued for a duration of 5 days. The culture result revealed *Alcaligenes Xylosoxidans*, a water-borne gram-negative bacteria. The patient was discharged on a 6-weeks course of oral Trimethoprim-Sulfamethoxazole. By day 10, the patient's inflammatory biomarkers had returned to normal levels. The splint was maintained in 25° of plantar flexion for 3 weeks then a pneumatic walking boot was applied maintaining the ankle in neutral position for another 3 weeks. Weight bearing with the boot was started after 6 weeks for a duration of 3 weeks before passive and active ankle flexion and extension was initiated. At 3 months, the patient was able to plantarly bend his ankle against maximal resistance with a successful standing heel-rise test (Fig. 5, Fig. 6). After a follow-up period of twenty months, the patient remained pain-free with no recurrence of symptoms and was able to bear weight without restrictions. Both early and late postoperative radiographs did not exhibit any signs of bone erosion or infection in the remaining calcaneal bone. The gentamicin-infused cement remained in place and intact without changes throughout the follow-up period.

## 3 Discussion

Chronic osteomyelitis presents significant challenges for both patients and physicians involved in treatment. Calcaneal osteomyelitis, which accounts for 3–10 % of all osteomyelitis cases [5], is commonly associated with traumatic events, surgical procedures, or diabetic ulcers [1]. Traditionally, the management of chronic calcaneal osteomyelitis (CO) has involved aggressive measures such as partial or total calcanectomy, or even below-knee amputation [6], all of which are associated with high mortality [7] and limited ambulation [8]. Therefore, more conservative approaches have been attempted, including extensive surgical debridement of necrotic and infected bone, soft tissue coverage, or prolonged courses of culture-specific antibiotics [2], with the aim to eradicate infection, preserve functionality, and achieve wound closure [9,10].

The underlying ischemic process in chronic osteomyelitis, characterized by necrosis and edema, hampers the effectiveness of systemic antibiotic delivery at their usual bactericidal levels [11]. Conversely, the controlled local release of antimicrobials through antibiotic-containing cement spacers has shown promise in achieving the high bone concentrations required for effective elimination of targeted organisms, while minimizing the risk of toxic serum levels [12,13].

Few reports advanced the concept of antibiotic-containing cement spacers in the management of well-organized osteomyelitis, such in the case of bone abscesses. This promising alternative to more radical options was described as effective and secure [[12], [13], [14], [15]]. Our case represents a particular chronic osteomyelitis, the pyogenic osteomyelitis or Brodie's abscess on a weight bearing bone. Filling the dead space following evacuation and debridement in a single stage surgery could be a challenge in this situation. We are aware of only one case of calcaneal abscess treated with surgical debridement and cement spacer for mechanical support, but with no mention of Achilles reconstruction [11].

This particular case involves a few unique technical considerations. The surgical procedure involves MRI-guided debridement, and there is no anchoring of the Achilles tendon to the bone. Typically, the surgical excision of infection is considered complete when there is visible bleeding [15]. Since the infected-healthy bone interface is often indistinct and might yield insufficient bone resection, an additional guidance from MRI images could be of real benefit intra-operatively. Additionally, apart from the contraindication in the presence of infection, there was no suitable location for anchoring the Achilles tendon. Following eight weeks of immobilization with a posterior splint, suturing the split borders of the Achilles tendon to the adjacent soft tissue structures successfully restored full active plantarflexion of the ankle joint during gait.

The most commonly used method for local antibiotic delivery is antibiotic-eluting PMMA cement. This is preferred due to its long-lasting effect and structural support. However, there have been some drawbacks observed with the use of PMMA cement. These include pathologic fractures [16], thermal damage to the antibiotics impregnated in the cement, and the need for surgical removal in some cases [17,18]. With a structure similar to the biological hydroxyapatite within bones, biodegradable synthetic carriers such as the calcium phosphate cement were advanced as a potential alternative to PMMA cement, as they preclude the need for removal and do not induce exothermic reactions [19,20]. However, their long-term mechanical performance was found to be poor [21].

## 4 Patient perspective

Patient was very satisfied, pain free and has returned to ambulation and daily activities.

## 5 Conclusion

Antibiotic-loaded cement has proven to be effective in the treatment of a calcaneal abscess that is deeply embedded within cancellous bone. This technique can be incorporated into the existing repertoire of conservative foot surgeries. By utilizing antibiotic-loaded cement, not only does it provide sustained local control of the infection, but it also offers essential mechanical support for walking and ambulation. This combination of infection management and mechanical stability contributes to a successful outcome in treating calcaneal abscesses while preserving the functionality of the foot.

## Informed consent

The patient was informed that data regarding the case would be used submitted for publication and provided consent.

## Ethical approval

The study was exempt from ethical approval in our institution, since it was based on blinded electronic chart only.

## Funding source

The authors of this study declare that there are no funding sources.

## Author contribution

Conceptualization: KY; Data curation: All authors; Formal analysis: All authors; Roles/Writing - original draft: KY, BA, JM, JO; Writing - review & editing; All authors.

## Guarantor

Kaissar Yammine.

## Research registration number

Our case report is not a First in Man’ study.

## Conflict of interest statement

The authors of this study declare that there are no conflicts of interest.
